# The Proteogenomic Mapping Tool

**DOI:** 10.1186/1471-2105-12-115

**Published:** 2011-04-22

**Authors:** William S Sanders, Nan Wang, Susan M Bridges, Brandon M Malone, Yoginder S Dandass, Fiona M McCarthy, Bindu Nanduri, Mark L Lawrence, Shane C Burgess

**Affiliations:** 1 Department of Biochemistry & Molecular Biology, Mississippi State University, MS, USA; 2 Department of Computer Science, University of Southern Mississippi, MS, USA; 3 Department of Computer Science & Engineering, Mississippi State University, MS, USA; 4 Department of Basic Sciences, College of Veterinary Medicine, Mississippi State University, MS, USA; 5 Institute for Genomics, Biocomputing, and Biotechnology, Mississippi State University, MS, USA

## Abstract

**Background:**

High-throughput mass spectrometry (MS) proteomics data is increasingly being used to complement traditional structural genome annotation methods. To keep pace with the high speed of experimental data generation and to aid in structural genome annotation, experimentally observed peptides need to be mapped back to their source genome location quickly and exactly. Previously, the tools to do this have been limited to custom scripts designed by individual research groups to analyze their own data, are generally not widely available, and do not scale well with large eukaryotic genomes.

**Results:**

The Proteogenomic Mapping Tool includes a Java implementation of the Aho-Corasick string searching algorithm which takes as input standardized file types and rapidly searches experimentally observed peptides against a given genome translated in all 6 reading frames for exact matches. The Java implementation allows the application to scale well with larger eukaryotic genomes while providing cross-platform functionality.

**Conclusions:**

The Proteogenomic Mapping Tool provides a standalone application for mapping peptides back to their source genome on a number of operating system platforms with standard desktop computer hardware and executes very rapidly for a variety of datasets. Allowing the selection of different genetic codes for different organisms allows researchers to easily customize the tool to their own research interests and is recommended for anyone working to structurally annotate genomes using MS derived proteomics data.

## Background

Expressed proteins provide experimental evidence that genes in the genome are being transcribed and translated to produce a protein product. Recently, a new structural genome annotation method, proteogenomic mapping, has been developed that uses identified peptides from experimentally derived proteomics data to identify functional elements in genomes and to improve genome annotation [1,2]. Initially used for the structural annotation of prokaryotic genomes, proteogenomic mapping is rapidly gaining traction in eukaryotic genome annotation projects with larger genomes as a complementary method [3,4].

Proteogenomic mapping can identify potential new genes or corrections to the boundaries of predicted genes by using peptide matches against the genome that do not match against the predicted proteome to generate expressed Protein Sequence Tags (ePSTs) [2]. When aligned with the genome and combined with the published structural annotation, these ePSTs are indicative of translation throughout the genome and can serve to supplement traditional structural genome annotation methods [3-5].

While a number of research groups are becoming increasingly active in the field of proteogenomic mapping [1-5], there is a lack of published and standardized tools to rapidly and exactly map identified peptides back to the genome translated in all 6 reading frames. To our knowledge, there is only one comparable tool, PepLine [6], which utilizes a de novo based spectral identification methodology. In contrast our tool is implemented to work with the output from LC MS/MS combined with database search based spectral identification algorithms. PepLine uses peptide sequence tags (PSTs), short spectral match translations of 3-4 amino acids with flanking matches on either end for searches against the genome, where our tool works with peptides derived from MS/MS databases searches. While PepLine's use of PSTs allows the direct searching of spectra against the genome, a staged search method of searching spectra identified against database searches is an alternative.

## Implementation

The Proteogenomic Mapping Pipeline is free to obtain and use, written completely in Java, and available for all common computer platforms. It is licensed under the GNU GPLv3 license making it completely open source and making the source code and implementation methodology available to the end user [7]. We have endeavored to make this tool as easy to use as possible and have provided both a command line version and a graphical user interface (GUI) for all common platforms.

### Data Input and Customization

**Figure 1:**
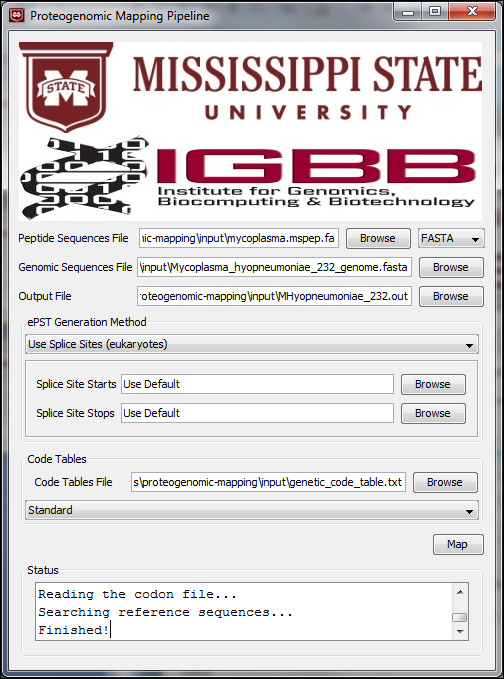
**Proteogenomic Mapping Pipeline Windows GUI**. The proteogenomic mapping pipeline requires three files from the user and offers several options. a. First, the user must provide a FASTA formatted file specifying the proteins for which to search. b. The user also supplies a FASTA formatted file specifying the genome in which to search for the peptides. The file can contain the entire genome as one large entry or multiple entries containing only selected features of interest. For example, the file may contain all exons for an organism. c. The user then selects an output file. Two files will be created. The file selected by the user will contain detailed information about the mapping. An additional FASTA file, with ".fasta" appended the name as the file selected by the user, contains the ePST sequence in FASTA format. d. The user can select to ignore splice sites or to use canonical splice sites when searching upstream for the start of the ePST sequence and downstream for the stop of the sequence. e. A genetic code table file, which specifies the mapping from codons to amino acids as well as start and stop codons, must also be provided. f. Because the code table file can contain multiple mappings, the desired mapping must be selected.

The GUI is shown in Figure 1 and takes as input from the user 3 files: a FASTA file of the peptides to be searched, a FASTA file containing the nucleic acid sequences the peptides are to be mapped against (typically the genome), and a file containing the genetic code to use based on the format of the National Center for Biotechnology Information's (NCBI) toolkit for genetic codes [8]. Furthermore, FASTA output from the splice site prediction tool GeneSplicer [9] can optionally be provided. If present, the splice sites given in that file are used instead of the default splice sites. The user is also required to provide a file name and location for the three output files that will be generated.

To generate the FASTA file of the peptides to be searched, it is expected that the user will have performed spectral matching for their MS dataset of interest against databases generated from both the proteome and the genome translated in all six reading frames and confirm these peptide identifications using a peptide validation strategy. After validation, the unique peptide identifications resulting from a database search against the genome that are not contained among the proteome peptide identifications should be used as the list of peptides to be searched.

The command line version of the Proteogenomic Mapping Pipeline allows the same inputs as the GUI to be specified as command line arguments and can be run on standard computer platforms (Windows, Linux, Unix, MacOS). An example of using the command line version of the program is included in the README file provided with the application.

The application translates the nucleotide database to protein in all 6 reading frames using the genetic code selected by the user (we provide the most common genetic codes from NCBI [8] which are represented in NCBI's standard format for genetic codes in the *genetic_code_table *file included with the application) and maps the peptides to the translated genome using the Aho-Corasick string searching algorithm to provide rapid and exact matches of peptides to the genome [10,11]. The Aho-Corasick string matching algorithm [10] quickly locates all occurrences of keywords within a text string. The algorithm consists primarily of two phases. In the first, a finite state machine is constructed from the set of keywords. The time to construct this machine and its memory requirements are linearly proportional to the sum of the lengths of the keywords. The second phase consists of running the state machine using the text string as input. This phase takes time linearly proportional to the length of the text string. Thus, the time to run the entire algorithm is proportional to the sum of the length of the keywords and the length of the text string. In our case, the peptides for which to search are the keywords, and the reference genome against which to search is the text string.

### ePST Generation

**Figure 2:**
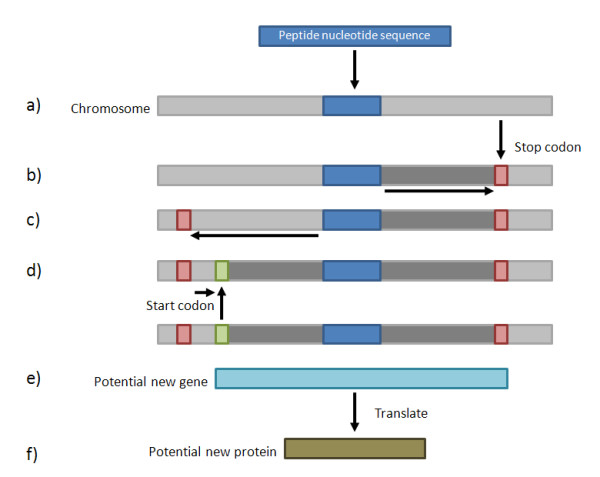
**Prokaryotic ePST Generation Process**. a. Map the peptide to the translated genome. b. Extend the mapped peptide in the 3' direction to an in-frame stop codon. c. Extend the mapped peptide in the 5' direction to an in-frame stop codon. d. From this 5' in-frame stop codon, proceed in a 3' direction to identify an in-frame start codon. e. Final ePST. f. Generate translated ePST sequence.

**Figure 3:**
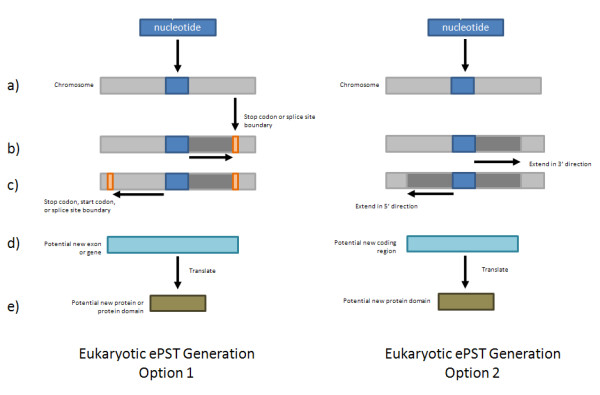
**Eukaryotic ePST Generation Process**. a. Options 1 & 2: Map the peptide to the translated genome. b. Option 1: Extend the mapped peptide in the 3' direction to an in-frame stop codon or splice site boundary. Option 2: Extend the mapped peptide in the 3' direction the number of codons selected by the user. c. Option 1: Extend the mapped peptide in the 5' direction to an in-frame stop codon or start codon, or splice site boundary. Option 2: Extend the mapped peptide in the 5' direction the number of codons selected by the user. d. Final ePST. e. Generate translated ePST sequence.

Once a peptide has been mapped to a nucleotide sequence, the reverse translated peptide is used to create an expressed Protein Sequence Tag (ePST) [2]. Figure 2 illustrates the ePST generation process for prokaryotes and Figure 3 shows both options for the ePST generation process in eukaryotes. For prokaryotes, the reverse translated peptide is extended in the 3' direction to an in-frame stop codon. In the 5' direction, the first in-frame stop-codon upstream of the peptide (5' stop) is identified and the peptide is extended to the first in-frame start downstream from this 5' stop before the start of the peptide. In the case that no in-frame start occurs between the 5' stop and the start of the peptide, the start of the peptide is used as the start of the ePST. The process is more complex for eukaryotes due to splicing. For eukaryotes, the peptides can be extended to produce ePSTs using three different approaches. In the first approach, the peptide is extended downstream to the first in-frame stop or splice site signal [12] and upstream until the first in-frame start, in-frame stop, or splice site signal. We have found that this approach often generates ePSTs that are far longer than typical exons. We speculate that this is because the potential new ORFs identified by this approach do not have a canonical splice site signal. While the application does default to using canonical splice site signals, our second approach includes the option of using predictions from GeneSplicer [9], a computational splice site prediction tool, by allowing the user to select to input GeneSplicer output for use instead of the canonical splice site signals. A third option is to extend the peptide upstream and downstream by a nucleotide length to be specified by the user.

### Output File Description

Three output files are produced by the application. The first file is a FASTA file containing the ePSTs generated for the dataset. The second file is a more detailed tab separated text file containing the original peptide's identification, the peptide sequence, the FASTA header for the nucleotide sequence containing the match, the mapping start and end locations for the reverse translated peptide, the strand the nucleotide match, the reading frame of the match, the reverse translated peptide sequence, a longer nucleotide sequence extending from the 5' in-frame stop codon immediately upstream of the peptide to the 3' in-frame stop codon immediately downstream of the peptide, the ePST nucleotide sequence and the start and stop locations of the ePST on the nucleotide sequence, the length of the ePST, and the translated ePST. The third file is a GFF3 file containing the ePSTs generated for the dataset to provide researchers with a file format they can quickly load into genome browsers for data visualization.

### Example Datasets

To test our implementation we acquired previously published proteogenomic mapping datasets for a number of organisms. For a relatively small example data set, we selected a proteogenomic mapping dataset for the channel catfish virus [5]. This small dataset contains 407 unique peptide identifications, of which 17 peptides did not map to the predicted proteome of the virus, but do map to novel open reading frames in the viral genome, and expression of several of these genes was confirmed by RT-PCR. Our example dataset consists of a FASTA file of these 17 peptides and the reference genome (NC_001493.1) for the channel catfish virus. For bacterial examples, proteomics datasets from three different microorganisms [13] were used to test our application. For a eukaryotic example, a previously published proteomics dataset generated from chicken serum was utilized for testing [14]. Table 1 details the number of unique peptides and the number of unique peptides mapping uniquely to the genomic database search contained in each of these five datasets.

**Table 1:** Example Dataset Statistics

	CCV	*H. somnus*	*M. haemolytica*	PMU	Chicken Serum
# unique peptides	407	958	1,755	675	1,447

# unique peptides mapping exclusively to genome	17	305	1,585	376	92

## Results and Discussion

The output from the Proteogenomic Mapping Pipeline matches the previously published results against our test dataset [5], and our output provides additional information that not only places the mapped peptides on the appropriate nucleotide strand but also includes the reading frame in which the match occurs. Table 2 gives a list of the peptides and corresponding ePSTs for this dataset. We have also successfully tested this tool for proteogenomic mapping in previously published bacterial [13] and eukaryotic datasets [2,14]. Table 3 provides runtime analysis for each of our five test datasets, and demonstrates that the Proteogenomic Mapping Tool scales well for increasingly large datasets.

**Table 2:** Channel Catfish Virus Peptides and ePSTs.

ID	Peptide	Reading Frame	ePST
Proteinase-1	NLDLLDNSTG	+1	CTGCTGACCAGGCTACTGTTTGTATGCACAATCTTGACCTTCTCGACAATTCCACTGGTGCTCCACAAGGGGATCTCACCGATCCAAGAGAAGATGGGTAGG

Proteinase-2	LMPCSMSS	+1	ATGATCCGGACGAGGTTCCTAGTTCGAAGAGAGGGCCTTCTCGATGTGGTCTCTCCCGGTGAACTCTTCTCCGGAGAACACGGGGTAATCACCCCCGGGACTGAACGATATAGACTCATGCCATGTTCCATGTCCTCTATTTGAT

Proteinase-3	PSPVSSHPLAASVSGPC	-1	GTGATTCTTCGTCTTTCCGAGCCCCGTATCGTCGCACCCATTAGCCGCTTCGGTGAGTGGACCTTGTGTCGCAGACATCTTCAAGACAAGCGATTGGTTCAGATGGTGGAATTGGAATGAATATTCGCGTATATTCACCAGTGTCTTTTAAT

Proteinase-4	MRELVSM	+3	TTGATGTTTTTGTTCCCGTCTCTATATCTTTATTCAGAGTCTGAACCAGTGACACTTAGATTGTTATCATATGATTTAAACCATGATAGGTCACCATCTGTAAATTCCTCATGGTTCATGATCCCGTGCTTGGCACATATCATTATCAGAAGGATGGCCTTCATCGACAGCTCCACTCTCTGGTGGTCTCTGTCACTCACCGGCGTGCCCGGGGTCGCGTATTCCACCGCCGTGTCTCTGTTCAAGACGGCGAGTTGGCCTCTGGGGATATCGGCCGCCGTGACGGTCAGGGAGTTGATGAGAGAACTGGTCTCCATGTCAGTGTTTAGTCTCTGGAAGATTTCCTCAGCGGACATCTCGGGTCCCGCTGCTAATGCGAGCCTCAGGGTTTCACGGGTAATCGATAGATGCACCCGCTTGTGGCTATGCCGGGCGGCCGGCCTCTTTCCTCGTACACGCGGGGTTGGTTTGGGTTCGGCCACGTGCGCGCCCCGGCGTTCCAGTAACGTAACCGGACGCCTCGAGGGGACCCGCGCGGGCTCGGGATCGGCCCCGATACCACCGGCCGGGACACCGATCAGTTCCAGTGGCCCGCCCGCAGACGGTGGGTCTTCGTCCTCGCTCTCTTCGCTCTCCTCCTCGCTCTCCTCCTCCTCGCCTCCACTCTCCGTCTCGCCCCCTTGTCTATCCTCCTCGTCCTCCTCTCGGCACACTCCATCTCCGCGGGTGCCGTTCGAGTCCGGCACCGGATCGACACTCTCATCGTCACCCGATTCCTCACTGCTGAGCTCACGACCACCGGCGTACGATCCGTGGTAGT

Proteinase-5	RNDIAESSCLVA	-1	TTGATGACGTCCCAGTTCGCCAGGTCGGGTCTCACCATCGAGAGAAACGACATCGCAGAATCCAGCTGTCTGGTCGCGACCATCGACTCCATGGCCTCGGCGAGACTCGTTCCTTGAT

Proteinase-6	ISRDSIPILF	+3	ATGCTGACACACACCACCCCGAACAAGGCTGTACGTATCAGGTGCATCAAACCCAGGATACTCGCGGGGGGTGTTCCGGGTGTAGCTCTCACTACATACCGAAATTTTCCGAGGTCGGAGAGGTCGCTGCAGCTGTTGTGCTTGGTGCCGGATTGTGTGGCCCCCTTACCGGTACTGTTGACAGTCAGCGTTCCGAACTCGGTGAATTCGGTACTGTTGTACACAGACCACAGGCAGTTGACAGGGAAGACCTTCCCGGGTTCTCTCTTTTCGGGTATCTCTAGGGATTCAATCCCAATCTTGTTCAACCACTCGATGAAGGTGGTGGGTCCCTGTTGGTTGTAGA

Proteinase-7	QAVVPMNTF	-2	CTGTGCGTCAGTTGCTGTAACTTGACATCCGGGTTATCGGTTGGTTTCACCGATAGATCGACCGTGAACGGACCCGGGGGTAAATCGGCGGGCGCGACCTGCAGGGCCGCTCCGCAAGCGGTCGTCCCCATGAATACGTTCGAGCATATCACCGCCACATGTGCGTCCTCGAGGTAGT

Proteinase-8	QLGDGPLGGGHVDHIPF	+3	ATGTAGATGACCATGTCCAACTTGAGAGGTCCAATGTCTACCCCCCGTGGGTCGTGGTACAGAATGTGTGTGTTGTACATGTTCGTTATGAAGTTGATTCCATTGTCTCGGAACGCGACGGCGAGCGAGATGAGTTGTTTCAGGATCACGGCCCCCAGGAGGGTTCCATCGTCCGTCTCGCCATCGAAGTTCAGCTCGGTGATGGACCTCTTGGCGGGGGTCACGTAGATCATATACCTTTTCGTGCCATGGGCGCCCAGGGTGTAGTGGAACACCGGTACCACATATGGGATAGATTTTTGGATCGCCTGGATCCCCTCCATCAAAGACTGTATCGCCTCGAAATCAATGGTCGGTTTCAGCTCGAGGTAGACGATGTCATACGTAGGGGAGAATTCGGGGGGCCTGTATACCCTGATCTCCTTCACGCCGTACTCTTTGGTGGCCACGACCGGGTACACGGAGACGAGGTCCCGGGGGGTGAGGGTTATCAGTTTCTTCGCGGTGTGATCATCGCCCATGTCTGCGCGCGCAAGCCATGGCATGTATAGC

Proteinase-9	ARDLPRRF	+2	CTGTGAACAAATATATCTTCGAAGTTTGCCGCGAGGGTACCGACGAGGTCCCGCACGCGATCTACCAAGACGGTTTCCAGGACGTGTCTCACGACTGGAAGGGCCGGGCCCGGCCATATCACCACGATCGAACCCGGGTCGAGCGCGCACTCGATAGA

Proteinase-10	EVVILQ	-1	ATGCGTACACCGCATACGCCTTCAGCACTGCACTGTCACGGCTCAGGTCCCATTTACGACGTGCGGGGTAAGGCCTGTCTCCCTTCAGAAATTGCGTGAGCTCGTAGTATTCGCTCAGCACCCTCTGTCCCAGGAACTGGCGTATCCGAGGACAACCACCCCTCGAATGGTACACGTGTTCGTCCAGGAAATCATCGACGAGCGTGAAGCGGATGACCTTGACACCGCAGTCTGGACACACGTGACGATCGCTCTTCACATCGTCCGGGATCAAACCTCCCTTGGGTCCGAAGTACAGTCTCGTCATGAACACGAGGTTGTCATCCTGCAAGGTACCGTGGGCGACTATTGTATCGTAATCCAAGGTAACATCGCAAAACCACACACCTCCGTCCGCACGCCATCCGCTTGGCTTGAGCATTCCCCTGGGTGCCGCGGACCATCTGAACCCCCTGTCCGTGGGGTTGCTGACCCGCTCACCGTCTGTCACCAGGGAACCGCATTCGAAATCCATCGCCTCAGTAGTGGATTGTCAGAGATCGTTCTATGGGTATCTGGTCAGTGTGAATTATTGGAATGGGCGCTCGCAGTATTCTTCAATCGTTCTTTTTCGGGCACCATGAGACTCTCGGGATCGAGGAAGCCGCCGGCGGTCCACCGGATGCGACACGTGAGATCCGATAACCTATAAA

Trypsine-1	IPFVSGLMNAQIILFSGPCMIGRNAAVSCK	+3	CTGACAGCCACGGAATCATCGGGGGTGTACACAACTTCCGAATCCACGGAGTCCATCACCGCGGTGGCGATCCCGACCATCGCACGGAGTTCGGCCTCGGTCCCGATCTTCTCAAGGAAAAAACCAAGGTGTTCCGGGTATACCTTTAGAATTCCCTTCGTTTCCGGGTTGATGAACGCGCAGATCATCTTGTTCTCGGGTCCTTGTATGATAGGCAGGAACGCGGCCGTCTCGTGCAAGCTATCGAAACGATCCATATCATGGGCACCGCCGATGAGATCCATCCCGATGTTCTTGCGGAGTGCATCCATTTCGCTCACAAGAAGATAAA

Trypsine-2	ARTVFLNVRPGWSR	+3	GTAGAGGAGGGCCCGAACCGTCTTCCTAAATGTGAGACCGGGGTGGTCTCGGAAGGACGACGCGTCAGTCGGGCAACCCGCCATCGTACCGGCCAAGAGGGACTTGACACAGGTGCGGATCATTCCCACCTTGTATCCGATCTGGATCGCCCCCTGTGTGACCTGGTTGGTCAGGCTCATGATCTGTTGGTACCACTGCTGGTACGCCATCACTTCCCCGAGGCGCTCGTTGGTCGTCGTACCCAGCTCCTTCAGGCCAGTAGCTAAACGCTTGAAGTTTGTATCCATGGCCAGCATCTGGAGGTTTATCTGGTTTTGTAGGTCGTCCACCCTCCCGTTCACTGCCCTGATGTTGCGGTCTAACTTGGCCGATATCAGGGCGATACTGTCTCCCAGTTCCGTGATCACATCCGCGGTCTTGTCCAATTGTGCCTGTAAACCGTCTATTTTGGAGGCTGCCAATGTGGCGACCGCCAGTGCTGCCGTCGACGCGACGAGTGCCGCGCTGGACATGGTTATCGCGGCCACCGATAACCCGAATTTATCGCTCGTGGGCACCGATCCGCCCGAGGCGGGCGCGAACATCTTAACCTTTTCGTGTTCGAGGTCCAGGTCAACGAGGCTATTTTTCAATAGTTCGTTACTCCGCTGGAAGTCCAGGAGTATCGCCGCCGTCTGAT

Trypsine-3	EGQAQRTCAYPSAGLLQASQGR	+3	CTGTGAAGCCGGCCGTGAGGGACAAGCGCAACGAACATGTGCCTACCCCAGCGCTGGTTTACTTCAAGCATCACAAGGCCGAGCTGGCCAAGGCGCTGGTTGAG

Trypsine-4	PCSRTSGSGACSGR	-1	CTGCGTAAGACGGAGGAGACCGTGCTCGCGGACGAGCGGTTCCGGGGCCTGCTCGGGCCGGAGATGGTGGCACGGCTATTGAA

Trypsine-5	NRTRVYTMPGWR	-2	TTGGGTATCAGCTTCCGTCCGCCCCCGGAGCCGCACTCGGGACACTCCCGGGGGGTGCAGAAGAAACAGAACACGTGTTTACACGATGCCCGGTTGGAGGGAAACACGGCCTCCCCCTGGCAGAAACAACACGGAAAGACTGGAGACATGAT

Trypsine-6	LKSPPGLRK	-1	CTGGAAAGGCTGAAAAGTCCACCGGGACTGCGAAAGTGAC

Trypsine-7	VARGEDATCPNDKGSEPR	+3	CTGGAAACAGAACTTCTCGAGGCCATCCGAGACGGTGTCGCGGGTGAAGAGTTTCGCGTGGCAGCCCCTCCGCGCGGGAATGACCACGACGCTGCACTCCAGTATCACCTCGTTGAGGCCCACATCGAGGGTTCCGAGGCTCAGTGCACATGGTATGTCCGCTTCCGTGAACGTCTCCACGCATCTCTTGCCGGTGTCCTCGGACTCATCTATCCCTCCTATCATGTTCAGGTAGACTCGGTCTTCCATGTGGACATGCCAGTAACCGAGGACCTTGCCCATGGGGATCTGGTGCGAGTACTTGAGCGTGCCCGGAGCGACCTGTACCATTGTTTGGTGGGCCTCGATCGGCTGCTGGTACTTGCGCATGTGCGCGGGGATGGAGGGGTCGTCGACCGGGTCGCCCGGGGAGAAGATGCAACATGTCCCAACGACAAAGGTTCCGAACCCCGTGAGTGGAACCTCGTATAGC

**Table 3:** Runtime Analysis For Example Datasets.

Dataset	Genome Size	# unique peptides mapping to genome	Runtime (ms)
CCV	0.1-Mb	17	563

*H. somnus*	2.3-Mb	305	2,932

*M. haemolytica*	2.8-Mb	1,515	4,507

PMU	2.5-Mb	201	3,003

Chicken Serum	1,050-Mb	92	127,991

Possible future updates to this application include parallelization of the searches against the genome in all 6 reading frames, and the introduction of better thread support to improve performance further on today's modern increasingly multi-core processors.

## Conclusions

The Proteogenomic Mapping Pipeline provides a standalone tool that facilitates a streamlined mapping of peptides to a target genome for structurally genome annotation through the use of proteomics. This software can be used on a variety of current operating systems and is its ability to use a variety of genetic codes makes it easily customizable for researchers performing proteogenomic mapping in a variety of prokaryotes, eukaryotes, and viruses.

## Availability and requirements

**Project name**: The Proteogenomic Mapping Pipeline

**Project home page**: http://www.agbase.msstate.edu/tools/pgm/

**Operating system(s)**: Windows XP, Vista (x86), Vista(x64), Linux, MacOS

**Programming languages**: Java

**Other requirements**: Java

**License**: GNU GPLv3 [7]

**Any restrictions to use by non-academics**: None

## Authors' contributions

FMM and SCB developed the initial procedure for generating ePSTs. BN and ML developed the algorithms for generating ePSTs for prokaryotes. SMB and NW designed the pipeline and NW implemented the initial Perl version of the pipeline. WSS and BMM implemented the current Java version and the graphical user interface. YSD implemented the string matching module. WSS and SMB drafted the document. BMM developed the software documentation. All authors read and approved the final manuscript.
